# Primary Carcinoid Tumor of the Testis

**DOI:** 10.1155/2015/687482

**Published:** 2015-02-11

**Authors:** Albert A. Petrossian, Joseph Habibi, David E. Rapp, Dharamdas Ramnani

**Affiliations:** ^1^Virginia Commonwealth University, Division of Urology, P.O. Box 980118, Richmond, VA 23298-0118, USA; ^2^Virginia Urology Center, 6900 Forest Avenue, Suite 200, Richmond, VA 23230, USA

## Abstract

Primary carcinoid tumors of the testis are a rare entity comprising less than 1% of all testicular neoplasms. Their presence should be considered particularly when evaluating a testicular lesion in an older male patient. Immunohistochemical studies may aid in diagnosis and radiographic evaluation is important to rule out metastatic origin. Primary carcinoid tumors of the testis are associated with an excellent prognosis; however, surveillance is important given rare reports of delayed metastases.

## 1. Introduction

Carcinoid tumors are the most commonly encountered small bowel malignancy and are believed to arise from neuroendocrine cells [1]. They are classically found at the tip of the vermiform appendix or the terminal ileum although they have also been described to occur in the lungs, pancreas, rectum, and genitourinary tract [2]. Carcinoid tumors of the testis are a rare entity comprising less than one percent of all testis tumors [3]. Carcinoid tumors of the testis can arise as a metastasis from an extratesticular primary, as a component of a teratoma, or as a primary tumor, with primary tumor being most common [4]. In this report we describe a case of a primary carcinoid tumor of the testis and literature review, with focus on presentation, diagnostic and pathologic evaluation, surgical management, and follow-up.

## 2. Case Presentation

**Figure 1:**
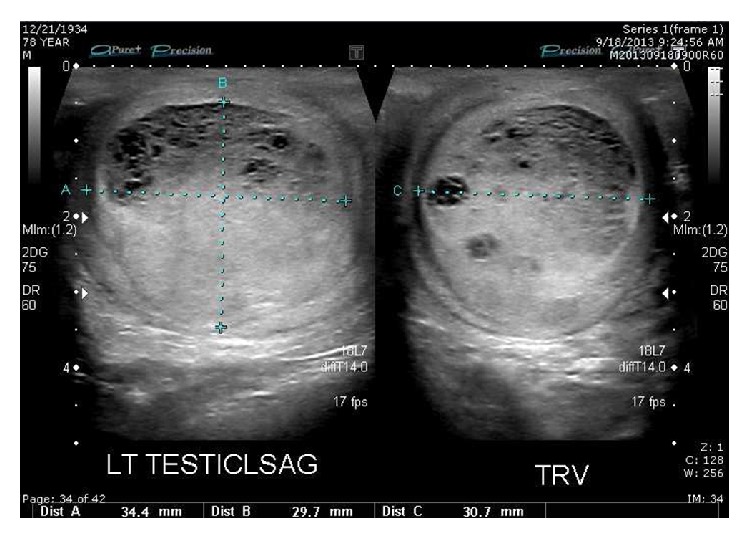
Scrotal ultrasound of left testis (sagittal and transverse views). Note the cystic and solid lesion of mixed echogenicity.

Patient GD was a 78-year-old Caucasian man referred with a four-month history of an enlarging, minimally tender left scrotal mass. He denied any weight loss, trauma, hematuria, or systemic symptoms. Physical exam revealed an indurated left testicle consistent with a mass. Urinalysis and microscopy were negative for infection. A scrotal ultrasound was performed which revealed a 3.4 × 3.0 cm well-circumscribed, hyperemic, cystic, and solid lesion of mixed echogenicity within the left testicle highly suspicious for malignancy (Figure 1). Tumor markers and staging CT scan were normal and demonstrated no evidence of metastasis. The patient underwent an uncomplicated left radical orchiectomy.

**Figure 2:**
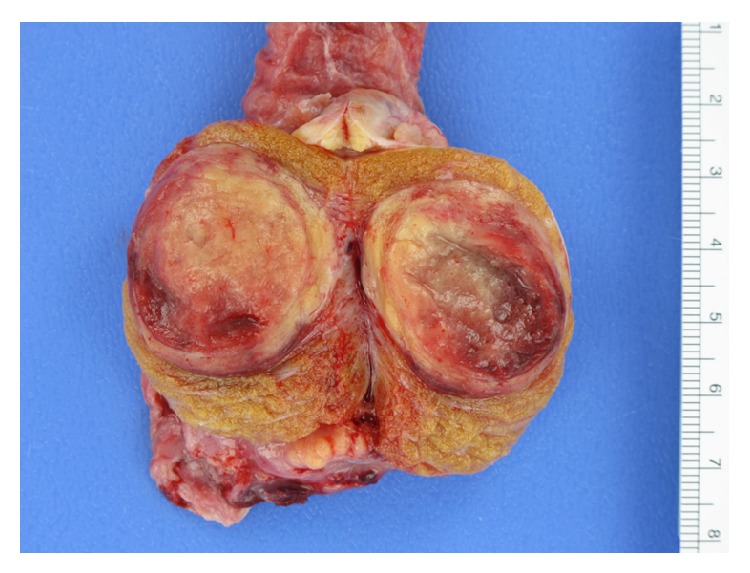
Gross specimen photograph of left testis showing a 3.4 cm well-circumscribed, yellow-tan mass with hemorrhagic areas.

**Figure 3:**
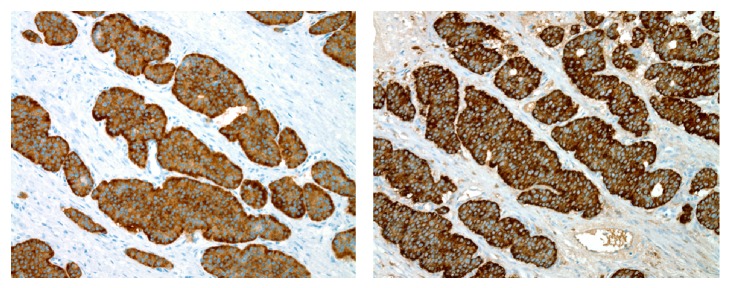
Positive synaptophysin and chromogranin staining of left testis carcinoid tumor.

Grossly, the orchiectomy specimen showed a 3.4 cm well-circumscribed, yellow-tan mass with hemorrhagic areas (Figure 2). The lesion was predominantly solid with an ill-defined cystic area filled with straw colored fluid. Histologically, the tumor had classic features of a carcinoid. The tumor cells were arranged in insular, glandular, and trabecular patterns in a background of fibrous stroma. The cells had abundant granular, eosinophilic cytoplasm and round to oval nuclei with a “salt and pepper” chromatin pattern. No teratomatous elements or other germ cell components were identified. A thorough sampling of the adjacent grossly normal testicular parenchyma showed only atrophic seminiferous tubules with no evidence of intratubular germ cell neoplasia. Immunostains for cytokeratin AE1/AE3 and neuroendocrine markers (chromogranin, synaptophysin, and CD56) were strongly positive supporting the diagnosis of a carcinoid tumor (Figure 3). The specimen did not appear to have any aggressive features such as multifocality, bilaterality, extratesticular extension, or evidence of vascular invasion. In addition, given its smaller size and lack of systemic symptoms, the risk of metastasis was determined to be low.

Given the lack of a defined protocol surrounding this entity, and its likely indolent course, the collective decision made proceeds with close observation consisting of periodic cross-sectional imaging. No biochemical follow-up is planned given absence of carcinoid symptoms on presentation and low-risk pathological features. At the time of this writing (12-month follow-up), the patient is without evidence of recurrence.

## 3. Discussion

The first reported case of primary testicular carcinoid was described by Simon et al. in 1954, with more than 50 cases reported at the time of this writing [4–12]. Although less frequent, additional cases of metastatic carcinoid to testis and carcinoid tumor associated with teratoma are reported. The origin of testicular carcinoid is unclear. They may arise as a result of differentiation of pluripotential germ cells to argentaffin-like cells or due to the development of a teratoma without any other teratomatous elements [13]. This hypothesis has been further reinforced by FISH analysis performed by Abbosh et al. which demonstrates the presence of classic genetic alterations that characterize germ cell tumors in these cells with carcinoid lesions: 12 p isochromosomy and overrepresentation [14].

Patients with testicular carcinoids are usually asymptomatic but may have testicular tenderness, hydrocele, or cryptorchidism on exam. Rarely, these individuals may experience carcinoid syndrome (episodic flushing, diarrhea, wheezing, and right-sided heart murmurs) as a result of the production of bioactive compounds such as serotonin, histamine, bradykinin, and prostaglandins [15]. Elevated serotonin levels and levels of its metabolite and 5-hydroxyindoleacetic acid (5-HIAA) are present in the blood and urine of those patients with this syndrome [16]. Traditionally, liver or lung metastases should be present to result in the manifestation of carcinoid syndrome [6].

In most cases, the standard work-up for a testicular tumor has already been performed preoperatively which may include serum tumor markers, and cross-sectional imaging to assess for lymphadenopathy. However, given the likelihood of an extratesticular primary, dedicated imaging of the lungs, abdomen and pelvis should be conducted with either plain films, chest/abdomen/pelvic CT, octreotide scintigraphy, video endoscopy, or a small bowel follow through [4]. Somatostatin receptor scintigraphy using indium-111 labelled octreotide is superior to CT in localization of primary tumor site and has a 96% sensitivity for detecting metastases. This unique imaging modality can identify about two-thirds of the primary and metastatic tumors. Octreotide binds to type 2 somatostatin receptors which are expressed by most carcinoid cells [6].

Radical orchiectomy is considered the treatment of choice as chemotherapy and radiotherapy are known to have little effect on carcinoid tumors of other origin [17]. Pathologically, the lesions are described as solid yellow-tan in appearance with an exceedingly firm texture due to striking desmoplasia which is characteristically present. Histologically, the neoplastic cells can form discrete islands, trabeculae, strands, glands, or undifferentiated sheets. Immunohistochemical studies show reactivity to antibodies to cytokeratin AE1 and AE3, chromogranin-A, neuron specific enolase, synaptophysin, and CD56 [15]. Much like pheochromocytomas, the lesions malignant potential cannot be predicted by its histological appearance [18]. Regardless, extensive testicular sampling should be performed to discover intratubular germ cell neoplasia, a minute teratoma, and/or evidence of a scar representing a burnt out or regressed germ cell component. Only once these steps have been performed can one comfortably anoint a tumor as a primary carcinoid [19].

A review of cases reported to date suggests that most cases primary testicular carcinoids have an excellent prognosis following orchiectomy. Due to the scarcity of this entity, data regarding prognostic indicators is lacking. However, literature review suggests that tumor size, invasion, and evidence of the carcinoid syndrome are associated with a worse prognosis and the development of metastases [20]. Stroosma and Delaere identified a mean tumor size of 70 mm versus 36 mm in patients with and without metastasis, respectively [4]. Similar findings are seen in gastrointestinal carcinoid tumors although, once again, the understanding of predictive features is complicated by the scarcity of these tumors [4]. The study of gastrointestinal carcinoid tumors has seen advances in genetic, molecular, and cellular sciences that may allow for the development of tumor markers to provide such predictive information [21]. Similar techniques may evolve to improve the understanding of testicular carcinoid; however, at the present time such markers are lacking.

The overall incidence of metastases is only 11% as seen in the literature, involving spread to lymph nodes, liver, skin, and the skeletal system [3, 19]. The vast majority of metastases are identified at the time of diagnosis and are seen in patients with high-risk pathologic features [4]. Rare cases of delayed metastases are reported, however, making surveillance necessary [7, 22]. Method of surveillance is poorly defined given absence of well-defined protocols. Literature review suggests significant variability to surveillance used in prior cases (chest radiography, CT, or 5-HIAA measurement), with many authors failing to provide information about follow-up protocol used. Nonetheless, we believe it is reasonable to perform annual physical examination coupled with abdominal and lung imaging as an initial surveillance protocol. However, until further data regarding optimal surveillance is available, we also believe that the decision regarding extent of surveillance should be made with the patient following an informed discussion. Serum levels of 5-HIAA or chromogranin A may be used however, this appears to be of most benefit in patients presenting initially with carcinoid syndrome.
